# Establishment of a Reproducible Ischemic Stroke Model in Nestin-GFP Mice with High Survival Rates

**DOI:** 10.3390/ijms222312997

**Published:** 2021-11-30

**Authors:** Hideaki Nishie, Akiko Nakano-Doi, Toshinori Sawano, Takayuki Nakagomi

**Affiliations:** 1 Institute for Advanced Medical Sciences, Hyogo College of Medicine, 1-1 Mukogawacho, Nishinomiya 663-8501, Japan; ds30046@hyo-med.ac.jp (H.N.); nakano@hyo-med.ac.jp (A.N.-D.); 2 Department of Therapeutic Progress in Brain Diseases, Hyogo College of Medicine, 1-1 Mukogawacho, Nishinomiya 663-8501, Japan; 3 Department of Biomedical Sciences, Ritsumeikan University, 1-1-1 Nojihigashi, Kusatsu 525-8577, Japan; t-sawano@fc.ritsumei.ac.jp

**Keywords:** ischemic stroke, nestin, neural stem/progenitor cells, green fluorescent protein, mouse model, C57BL/6 background, CB-17 background

## Abstract

An accumulation of evidence shows that endogenous neural stem/progenitor cells (NSPCs) are activated following brain injury such as that suffered during ischemic stroke. To understand the expression patterns of these cells, researchers have developed mice that express an NSPC marker, Nestin, which is detectable by specific reporters such as green fluorescent protein (GFP), i.e., Nestin-GFP mice. However, the genetic background of most transgenic mice, including Nestin-GFP mice, comes from the C57BL/6 strain. Because mice from this background strain have many cerebral arterial branches and collateral vessels, they are accompanied by several major problems including variable ischemic areas and high mortality when subjected to ischemic stroke by occluding the middle cerebral artery (MCA). In contrast, CB-17 wild-type mice are free from these problems. Therefore, with the aim of overcoming the aforementioned defects, we first crossed Nestin-GFP mice (C57BL/6 background) with CB-17 wild-type mice and then developed Nestin-GFP mice (CB-17 background) by further backcrossing the generated hybrid mice with CB-17 wild-type mice. Subsequently, we investigated the phenotypes of the established Nestin-GFP mice (CB-17 background) following MCA occlusion; these mice had fewer blood vessels around the MCA compared with the number of blood vessels in Nestin-GFP mice (C57BL/6 background). In addition, TTC staining showed that infarcted volume was variable in Nestin-GFP mice (C57BL/6 background) but highly reproducible in Nestin-GFP mice (CB-17 background). In a further investigation of mice survival rates up to 28 days after MCA occlusion, all Nestin-GFP mice (CB-17 background) survived the period, whereas Nestin-GFP mice (C57BL/6 background) frequently died within 1 week and exhibited a higher mortality rate. Immunohistochemistry analysis of Nestin-GFP mice (CB-17 background) showed that GFP^+^ cells were mainly obverted in not only conventional neurogenic areas, including the subventricular zone (SVZ), but also ischemic areas. In vitro, cells isolated from the ischemic areas and the SVZ formed GFP^+^ neurosphere-like cell clusters that gave rise to various neural lineages including neurons, astrocytes, and oligodendrocytes. However, microarray analysis of these cells and genetic mapping experiments by Nestin-CreERT2 Line4 mice crossed with yellow fluorescent protein (YFP) reporter mice (Nestin promoter-driven YFP-expressing mice) indicated that cells with NSPC activities in the ischemic areas and the SVZ had different characteristics and origins. These results show that the expression patterns and fate of GFP^+^ cells with NSPC activities can be precisely investigated over a long period in Nestin-GFP mice (CB-17 background), which is not necessarily possible with Nestin-GFP mice (C57BL/6 background). Thus, Nestin-GFP mice (CB-17 background) could become a useful tool with which to investigate the mechanism of neurogenesis via the aforementioned cells under pathological conditions such as following ischemic stroke.

## 1. Introduction

The central nervous system (CNS) includes neural stem/progenitor cells (NSPCs) that are derived from neuroepithelial cells. During early brain development, neuroepithelial cells produce radial glia, which later give rise to NSPCs localized in the subventricular zone (SVZ) of the adult brain [1]. Under pathological conditions, such as after ischemic stroke, SVZ-derived NSPCs that express the NSPC marker Nestin are activated and migrate toward the ischemic areas [2]. However, accumulating evidence shows that the migratory capacity of SVZ-derived NSPCs is limited [3,4]. In addition to these conventional neurogenic zones, NSPCs are increasingly being shown to be regionally activated within and around the ischemic areas [5,6]. Consistent with these findings, we previously showed that, although mature neural cells within the ischemic areas undergo cell death, Nestin^+^ NSPCs and/or NSPC-like cells with neurogenic potential develop within and around the ischemic areas in the brains of adult mice and humans [7,8,9,10,11,12,13,14]. Thus, it should be possible to promote neural regeneration by utilizing these endogenous NSPCs. However, to achieve this, it will be necessary to understand the exact fate of NSPCs over a long period under pathological conditions such as those that arise after stroke.

To determine the expression patterns of endogenous NSPCs, transgenic mice that express green fluorescent protein (GFP) under the control of the Nestin promoter have been used, i.e., Nestin-GFP mice [15,16]. However, the genetic background of most transgenic mice, including Nestin-GFP mice, comes from the C57BL/6 strain; stroke models of this strain of mice have several critical problems. We previously showed that C57BL/6 wild-type mice have more cerebral arterial branches and collateral vessels than are found in CB-17 wild-type mice. Furthermore, following middle cerebral artery occlusion (MCAO), the ischemic areas vary among individual C57BL/6 wild-type mice but are highly reproducible in CB-17 wild-type mice [10,11,17]. In addition, compared with C57BL/6 wild-type mice, we showed that CB-17 wild-type mice could survive for a longer period after MCAO and exhibited lower mortality [10,11,17]. These findings suggest that, compared with Nestin-GFP mice that have a C57BL/6 background [hereafter, “Nestin-GFP mice (C57BL/6 background)”], Nestin-GFP mice with a CB-17 background [hereafter, “Nestin-GFP mice (CB-17 background)”] would be more useful for investigating the fate of endogenous NSPCs following MCAO.

In the present study, we developed Nestin-GFP mice (CB-17 background) by backcrossing Nestin-GFP mice (C57BL/6 background) with CB-17 wild-type mice. We then investigated the traits of the newly generated Nestin-GFP mice (CB-17 background) as well as the expression patterns of GFP^+^ NSPCs over various periods, including acute, subacute, and chronic periods, following MCAO.

## 2. Results

### 2.1. Nestin-GFP Mice (CB-17 Background) Exhibit Fewer Artery Branches around the MCA When Compared with Nestin-GFP Mice (C57BL/6 Background)

**Figure 1:**
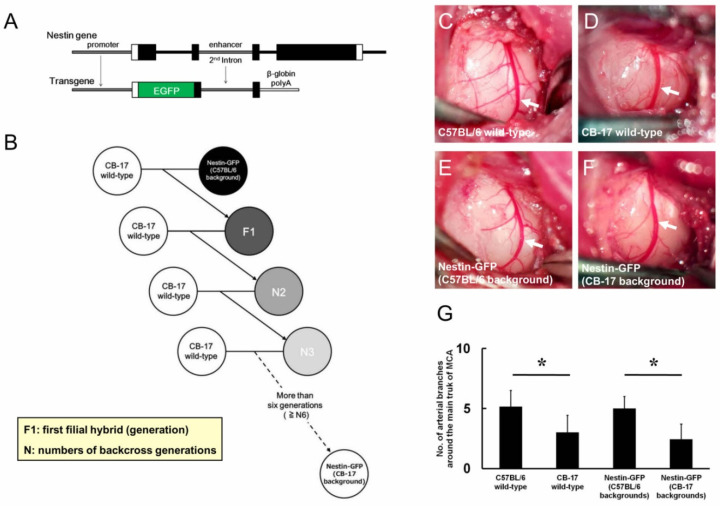
Genetic backgrounds of Nestin-GFP mice (C57BL/6 background) (**A**) and Nestin-GFP mice (CB-17 background) (**B**). Nestin-GFP mice (C57BL/6 background) carried the transgene under regulation of the *Nestin* gene promoter and second intron enhancer (**A**). Nestin-GFP mice (C57BL/6 background) were first crossed with CB-17 wild-type mice to generate F1 mice. Heterozygous progeny of the F1 mice were subsequently backcrossed with CB-17 wild-type mice for more than six generations (≥N6). The generated mice were termed Nestin-GFP mice (CB-17 background) (**B**). Comparative analysis of the arterial branches around the main trunk of the MCA (arrows, **C**–**F**). C57BL/6 wild-type mice exhibited higher artery branches than those of CB-17 wild-type mice (**C**,**D**,**G**). There were no significant differences in the numbers of artery branches among mice of the same background [C57BL/6 wild-type mice vs. Nestin-GFP mice (C57BL/6 background) (**C**,**E**,**G**); CB-17 wild-type mice vs. Nestin-GFP mice (CB-17 background) (**D**,**F**,**G**)]. Nestin-GFP mice (CB-17 background) had fewer artery branches compared with those of Nestin-GFP mice (C57BL/6 background) (**E**–**G**). * *p* < 0.05 among the four groups of mice [C57BL/6 wild-type mice, CB-17 wild-type mice, Nestin-GFP mice (C57BL/6 background), and Nestin-GFP mice (CB-17 background)] (**G**) (n = 7 for each group). Abbreviations: GFP, green fluorescent protein; MCA, middle cerebral artery; F1, first filial hybrid (generation); N, number of backcross generations.

We used Nestin-GFP mice (C57BL/6 background) that carried EGFP under the regulation of the second intron enhancer of the *Nestin* gene (Figure 1A). Nestin-GFP mice (C57BL/6 background) were crossed with CB-17 wild-type mice to generate first hybrid mice (F1), which were further backcrossed with CB-17 wild-type mice. By repeating this procedure, backcrossed mice (N2, N3, N4, N5, N6, etc.) were generated, and the skin of Nestin-GFP mice (C57BL/6 background) changed from black to white after more than four generations (≥N4), which indicated that they were Nestin-GFP mice (CB-17 background); specifically, we used backcrossed mice of more than six generations (≥N6) as Nestin-GFP mice (CB-17 background) (Figure 1B). In an investigation of the artery branches around the main trunk of the middle cerebral artery (MCA), C57BL/6 wild-type mice had a higher number of artery branches (Figure 1C) when compared with CB-17 wild-type mice (Figure 1D). Nestin-GFP mice (C57BL/6 background) also had many artery branches (Figure 1E), whereas Nestin-GFP mice (CB-17 background) exhibited fewer artery branches than were observed in Nestin-GFP mice (C57BL/6 background) (Figure 1F). The number of artery branches around the main trunk of the MCA was not significantly different between mice from the same background e.g., CB-17 wild-type mice versus Nestin-GFP mice (CB-17 background) and C57BL/6 wild-type mice versus Nestin-GFP mice (C57BL/6 background); however, the number was significantly lower in not only wild-type mice (CB-17 wild-type mice versus C57BL/6 wild-type mice) but also Nestin-GFP mice [Nestin-GFP mice (CB-17 background) versus Nestin-GFP mice (C57BL/6 background)] with a CB-17 background relative to those with a C57BL/6 background (Figure 1G).

### 2.2. Infarct Volume Is Reproducible in Nestin-GFP Mice (CB-17 Background) Compared with That in Nestin-GFP Mice (C57BL/6 Background)

**Figure 2:**
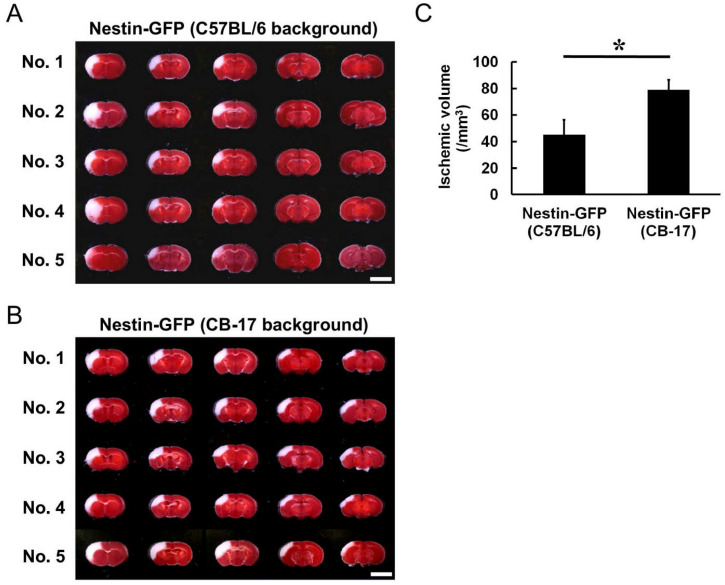
TTC staining of Nestin-GFP mice (C57BL/6 background) (**A**) and Nestin-GFP mice (CB-17 background) (**B**) tissue at 1 day after MCAO. The ischemic areas of Nestin-GFP mice (C57BL/6 background) were rarely observed in the posterior regions of the cortex; they occasionally ranged from the cortex to the striatum (No. 2 and No. 4 in **A**). In contrast, Nestin-GFP mice (CB-17 background) exhibited reproducible ischemic areas, which ranged from the anterior to the posterior cortex regions (**B**). Based on these results, the infarct volume of Nestin-GFP mice (C57BL/6 background) was significantly smaller than that of Nestin-GFP mice (CB-17 background), and the coefficient of variation was greater in Nestin-GFP mice (C57BL/6 background) than that in Nestin-GFP mice (CB-17 background) (**C**). Scale bars: 5 mm (**A**,**B**). * *p* < 0.05 between the two groups of mice [Nestin-GFP mice (C57BL/6 background) vs. Nestin-GFP mice (CB-17 background)] (**C**) (*n* = 5 for each group). Abbreviations: GFP, green fluorescent protein; TTC, 2,3,5-triphenylteterazolium; MCAO, middle cerebral artery occlusion.

To confirm that Nestin-GFP mice that originally had the C57BL/6 background had acquired the features of CB-17 mice after backcrossing for ≥N6, we investigated infarct volume between Nestin-GFP (C57BL/6 background) and Nestin-GFP (CB-17 background) mice. 2,3,5-triphenylteterazolium (TTC) staining was performed at 1 day poststroke; it showed that the ischemic areas were rarely observed in the posterior regions of the cortex in Nestin-GFP mice (C57BL/6 background) (Figure 2A), whereas the ischemic areas ranged from the anterior to the posterior regions of the cortex in Nestin-GFP mice (CB-17 background) (Figure 2B). The difference was reflected in the ischemic volume, which was significantly lower in Nestin-GFP mice (C57BL/6 background) than in Nestin-GFP mice (CB-17 background) (Figure 2C). In addition, the ischemic areas were variable among individuals in Nestin-GFP mice (C57BL/6 background); they occasionally ranged from the cortex to the striatum (coefficient of variation = 0.247; Figure 2A,C). In contrast, the ischemic areas were reproducible in Nestin-GFP mice (CB-17 background) (coefficient of variation = 0.096; Figure 2B, C). These results are consistent with those of a previous study in which ischemic areas were variable among C57BL/6 wild-type mice but highly reproducible among CB-17 wild-type mice [17].

### 2.3. Nestin-GFP Mice (CB-17 Background) Have Higher Survival Rates Than Those of Nestin-GFP Mice (C57BL/6 Background)

**Figure 3:**
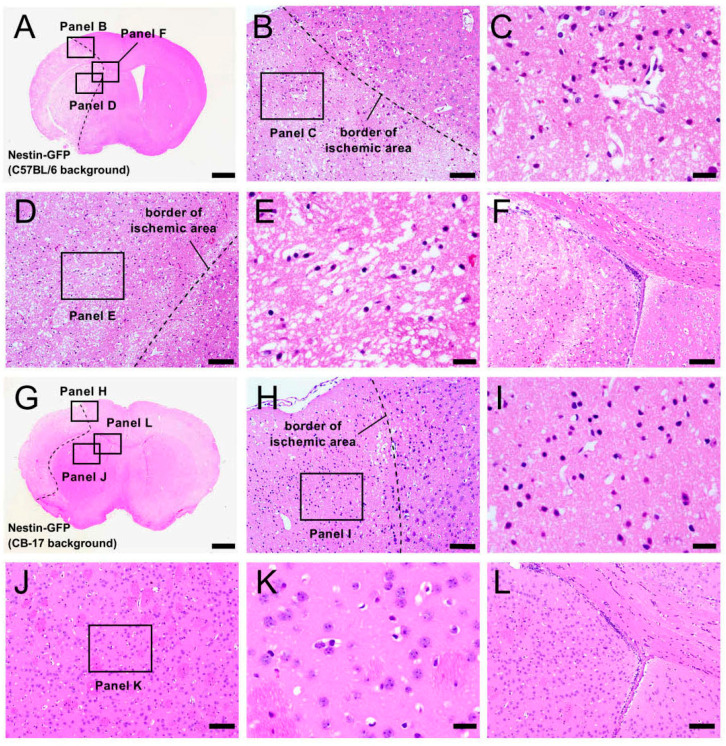

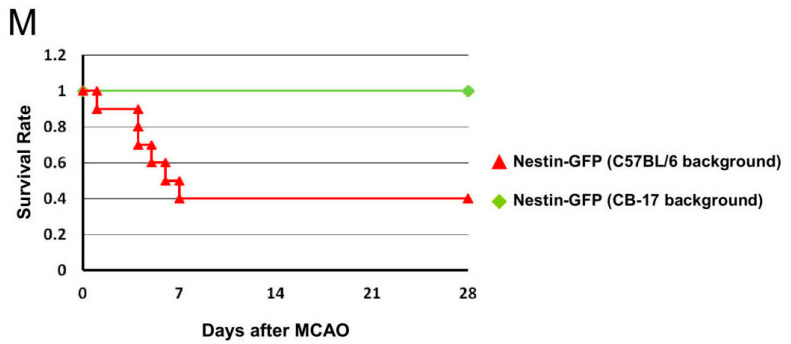
H&E staining of brain sections from Nestin-GFP mice (C57BL/6 background) (**A**–**F**) and Nestin-GFP mice (CB-17 background) (**G**–**L**) at 1 day after MCAO. In some Nestin-GFP mice (C57BL/6 background), the ischemic areas ranged from the ipsilateral cortex of the MCA region (**A**–**C**) to the striatum (**A**,**D**,**E**) near the SVZ (**A**,**F**). In contrast, in Nestin-GFP mice (CB-17 background), the ischemic areas were localized within the ipsilateral cortex of the MCA region (**G**–**I**), and the regions of the striatum (**G**,**J**,**K**) and SVZ (**G**,**L**) were intact. Survival rates are displayed using a Kaplan–Meier curve; they were significantly higher in Nestin-GFP mice (CB-17 background) than in Nestin-GFP mice (C57BL/6 background) (*n* = 10 per group) (**M**). Scale bars: 1 mm (**A,G**), 100 μm (**B**,**D**,**F**,**H**,**J**,**L**), and 25 µm (**C**,**E**,**I**,**K**). Abbreviations: H&E, hematoxylin and eosin; GFP, green fluorescent protein; MCAO, middle cerebral artery occlusion; SVZ, subventricular zone.

To study the histology of Nestin-GFP mice during the acute period, hematoxylin and eosin (H&E) staining was performed in Nestin-GFP mice at 1 day poststroke. Consistent with the TTC staining results (Figure 2A), the ischemic areas of some Nestin-GFP mice (C57BL/6 background) ranged from the ipsilateral cortex of the MCA regions (Figure 3A–C) to the striatum (Figure 3A,D,E) around the SVZ (Figure 3A,F). In contrast, but similar to the TTC staining results (Figure 2B), the ischemic areas of Nestin-GFP mice (CB-17 background) were localized within the ipsilateral cortex of the MCA regions (Figure 3G–I), and the regions of the striatum (Figure 3G,J,K) and the SVZ (Figure 3G,L) were intact.

We also compared the survival rates between Nestin-GFP mice (CB-17 background) and Nestin-GFP mice (C57BL/6 background) for up to 28 weeks. All Nestin-GFP mice (CB-17 background) survived this period (10/10 mice: 100% survival rate); however, Nestin-GFP mice (C57BL/6 background) frequently died within 1 week after MCAO (6/10 mice): only four of ten mice survived for 28 weeks (40% survival rate). These results are shown in the Kaplan–Meier curve in Figure 3M. A logrank test also showed that survival rates were significantly higher in Nestin-GFP mice (CB-17 background) than those in Nestin-GFP mice (C57BL/6 background) (*p* = 0.0046).

Taking the TTC staining and histology results together, Nestin-GFP mice (C57BL/6 background) presumably died more often due to events that occasionally occurred in the brains (e.g., brain herniation) and by larger-sized brain injury/infarction. Overall, Nestin-GFP mice (CB-17 background) appear to have increased survival rates with lower mortality when compared with those of Nestin-GFP mice (C57BL/6 background).

### 2.4. Expression Patterns of GFP^+^ Cells in Nestin-GFP Mice (CB-17 Background) Following Ischemic Stroke

**Figure 4:**
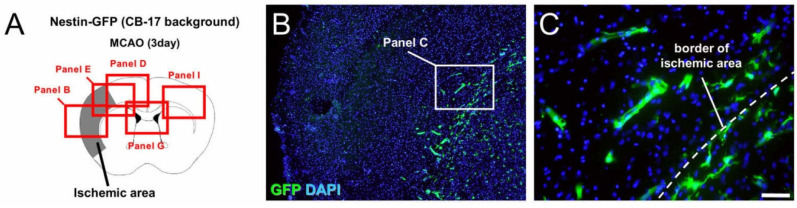

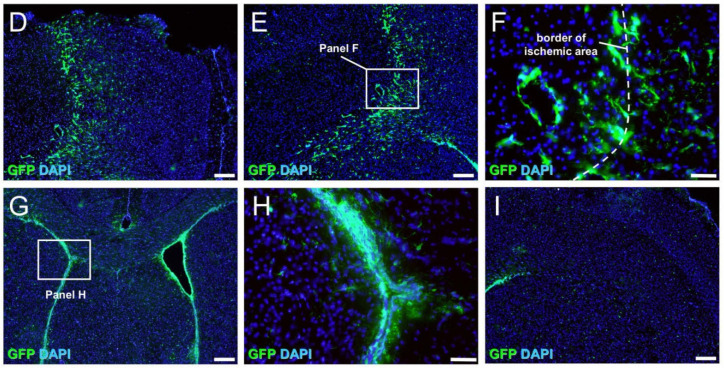
Immunohistochemistry for GFP at 3 days after MCAO (**A**–**I**). GFP was distributed at the site of the ischemic areas, including the peri-ischemic areas (**B**–**F**), as well as in the SVZ (**G**,**H**), whereas GFP was rarely observed in the contralateral cortex (**I**). [GFP (**B**–**I**: green), DAPI (**B**–**I**: blue)]. Results are representative of three replicates. Scale bars: 200 µm (**B**,**D**,**E**,**G**,**I**) and 50 µm (**C**,**F**,**H**). Abbreviations: DAPI, 4′,6-diamidino-2-phenylindole; GFP, green fluorescent protein; MCAO, middle cerebral artery occlusion; SVZ, subventricular zone.

In an investigation of the localization of GFP^+^ cells in Nestin-GFP mice (CB-17 background) following ischemic stroke, GFP^+^ cells were strongly expressed in the SVZ of sham-operated mice (Supplementary Figure S1). However, on day 3 after ischemic stroke (Figure 4A–I), GFP expression was observed at the site of the ischemic areas, including in the peri-ischemic areas (Figure 4B–F) as well as in the SVZ (Figure 4G,H). In contrast, GFP was rarely observed in the contralateral cortex (Figure 4I).

**Figure 5:**
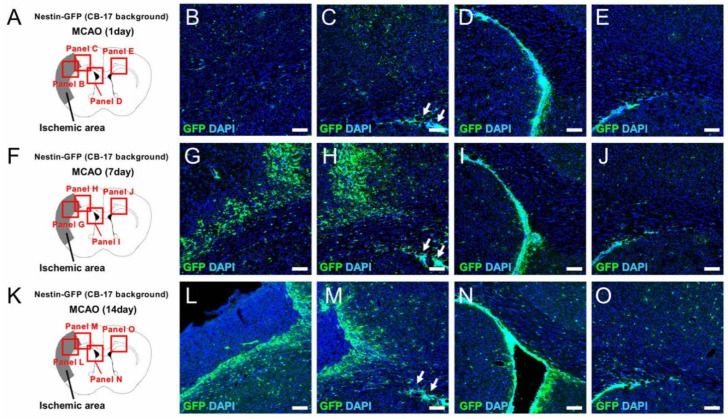

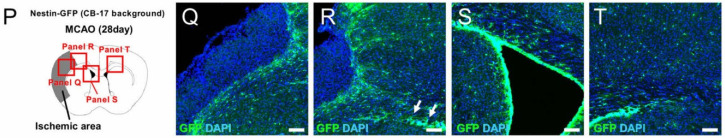
Immunohistochemistry for GFP at 1 (**A**–**E**), 7 (**F**–**J**), 14 (**K**–**O**), and 28 (**P**–**T**) days after MCAO. Although small numbers of GFP^+^ cells were observed within and around the ischemic areas at 1 day poststroke (**B**,**C**), many GFP^+^ cells were observed within and around the ischemic areas at 7 days poststroke (**G**,**H**). However, GFP^+^ cells within the ischemic areas were decreased at 14 days poststroke (**L**,**M**) and 28 days poststroke (**Q**,**R**), whereas GFP^+^ cells around the ischemic areas were observed at 14 days poststroke (**L**,**M**) and 28 days poststroke (**Q**, **R**). GFP^+^ cells were observed in the SVZ at 1—(**D**), 7—(**I**), 14—(**N**), and 28 days poststroke (**S**). Although some GFP^+^ cells from the ipslilateral side of the SVZ likely migrated toward the ischemic areas, it seems that they did not reach these areas at 1—(arrows; **C**), 7—(arrows; **H**), 14—(arrows; **M**), and 28 days poststroke (arrows; **R**). GFP^+^ cells were observed at the contralateral side of the SVZ but they were restricted in situ at 1—(**E**), 7—(**J**), 14—(**O**), and 28 days poststroke (**T**). [GFP (**B**–**E**,**G**–**J**,**L**–**O**,**Q**–**T**: green), DAPI (**B**–**E**,**G**–**J**,**L**–**O**,**Q**–**T**: blue)]. Results are representative of three replicates. Scale bars: 100 µm (**B**–**E**,**G**–**J**,**L**–**O**,**Q-T**). Abbreviations: DAPI, 4′,6-diamidino-2-phenylindole; GFP, green fluorescent protein; MCAO, middle cerebral artery occlusion; SVZ, subventricular zone.

The expression patterns of GFP^+^ cells were determined at various time points and for up to 4 weeks after ischemic stroke (Figure 5A–T). Immunohistochemistry showed that GFP^+^ cells were mainly found at the ischemic areas, including the peri-ischemic areas and in the SVZ, at 1—(Figure 5A–E), 7—(Figure 5F–J), 14—(Figure 5K–O), and 28 days (Figure 5P–T) poststroke. Immunohistochemistry also showed that GFP^+^ cells in the contralateral side of the SVZ were located in situ at 1—(Figure 5E), 7—(Figure 5J), 14—(Figure 5Q), and 28 days (Figure 5T) poststroke. Although GFP^+^ cells in the ipsilateral side of the SVZ migrated toward the ischemic areas, it is likely that they did not reach the ischemic areas at 1—(Figure 5C,D), 7—(Figure 5H,I), 14—(Figure 5M,N), and 28 days (Figure 5R,S) poststroke.

### 2.5. Traits of GFP^+^ Cells in Nestin-GFP Mice (CB-17 Background) Following Ischemic Stroke

**Figure 6:**
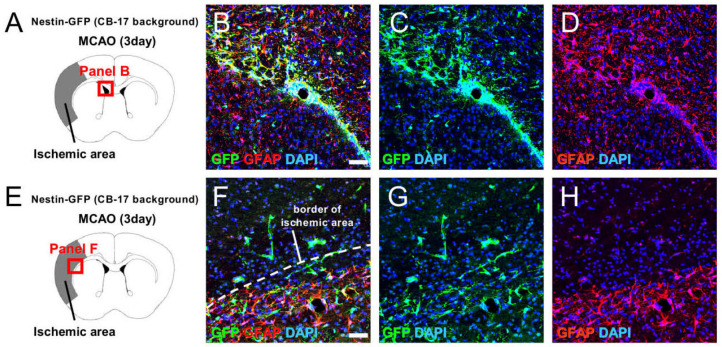

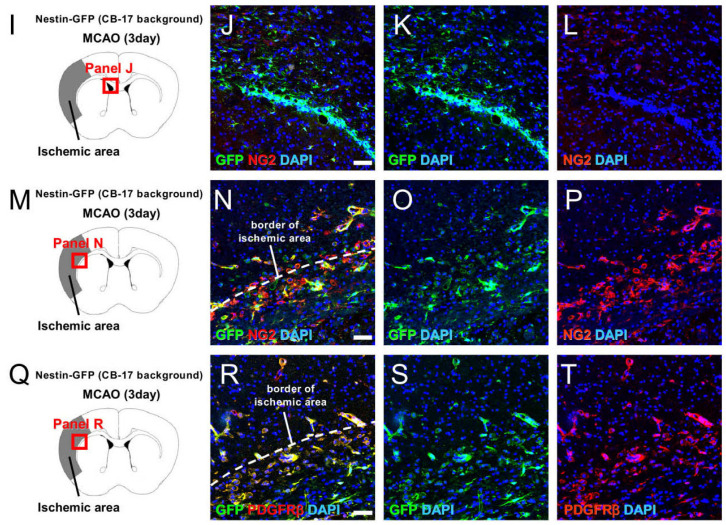
Double immunohistochemistry for GFP/GFAP (**A**–**H**), GFP/NG2 (**I**–**P**), and GFP/PDGFRβ (**Q**–**T**) at 3 days after MCAO. Some GFP^+^ cells in the SVZ (**B**–**D**) and at the peri-ischemic areas (**F**–**H**) expressed GFAP. Although GFP^+^ cells in the SVZ rarely expressed the pericytic marker NG2 (**J**–**L**), GFP^+^ cells within and around the ischemic areas expressed pericytic markers including NG2 (**N**–**P**) and PDGFRβ (**R**–**T**). [GFP (**B**,**C**,**F**,**G**,**J**,**K**,**N**,**O**,**R**,**S**: green), GFAP (**B**,**D**,**F**,**H**: red), NG2 (**J**,**L**,**N**,**P**: red), PDGFRβ (**R**,**T**: red), DAPI (**B**–**D**,**F**–**H**,**J**–**L**,**N**–**P**,**R**–**T**: blue)]. Results are representative of three replicates. Scale bars: 50 µm (**B**,**F**,**J**,**N**,**R**). Abbreviations: DAPI, 4′,6-diamidino-2-phenylindole; GFAP, glial fibrillary acidic protein; GFP, green fluorescent protein; MCAO, middle cerebral artery occlusion; NG2, neural/glial antigen 2; PDGFRβ, platelet-derived growth factor receptor-beta; SVZ, subventricular zone.

To investigate the traits of GFP^+^ cells following ischemic stroke, double immunohistochemistry was performed using brain sections at 3 days poststroke. Immunohistochemistry showed that some GFP^+^ cells in the SVZ expressed GFAP (Figure 6A–D; Supplementary Figure S2), indicating that GFP was expressed in the astrocytic population that is an origin of NSPCs in the SVZ [3,4]. In addition, some GFP^+^ cells at the peri-ischemic areas expressed GFAP (Figure 6E–H; Supplementary Figure S2), indicating that GFP was also expressed in reactive astrocytes that are also considered an origin of NSPCs [5,6]. However, most GFP^+^ cells within the ischemic areas did not express GFAP, indicating that they are likely to be other stem cell populations. We previously showed that brain pericytes acquire the traits of NSPC-like cells following stroke [7,10,18]; thus, we further investigated whether GFP^+^ cells express pericytic markers. GFP^+^ cells in the SVZ rarely expressed NG2 (Figure 6I–L; Supplementary Figure S2); however, GFP^+^ cells in and around the ischemic areas did express pericytic markers including NG2 (Figure 6M–P; Supplementary Figure S2) and PDGFRβ (Figure 6Q–T). These results suggest that GFP is also expressed in NSPC-like cells that likely originate from brain pericytes.

### 2.6. Neural Stem Cell Activity of GFP^+^ Cells from the Ischemic Areas and the SVZ

**Figure 7:**
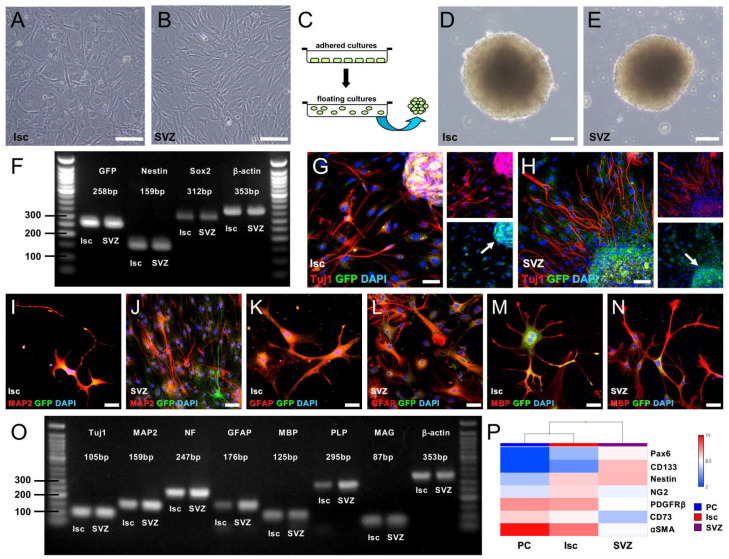
Adhered cells emerged from the ischemic areas (Isc) (**A**) and the ipsilateral SVZ (**B**) after incubation. Using floating cultures (**C**), both cells formed neurosphere-like cell clusters (**D**,**E**). According to RT-PCR analysis, cells from the Isc and the ipsilateral SVZ displayed not only GFP but also NSPC markers including Nestin and Sox2 (**F**). Even after differentiation, GFP expression was strongly retained in cell clusters (arrows, (**G**,**H**)), whereas the GFP signal was weak in differentiated cells with a neural shape (**G**,**H)**. GFP^+^ cell clusters from the Isc (**G**,**I**,**K**,**M**) and the SVZ (**H**,**J**,**L**,**N**) differentiated into Tuj1^+^ (**G**,**H**) and MAP2^+^ neurons (**I**,**J**), GFAP^+^ astrocytes (**K**,**L**), and MBP^+^ oligodendrocytes (**M**,**N**). [GFP (**G**–**N**: green), Tuj1 (**G**,**H**: red), MAP2 (**I**,**J**: red), GFAP (**K**,**L**: red), MBP (**M**,**N**: red), DAPI (**G**–**N**: blue)]. RT-PCR analysis also confirmed that these cells expressed various neural markers, including neurons (Tuj1, MAP2, and NF), astrocytes (GFAP), and oligodendrocytes (MBP, PLP, and MAG) (**O**). Heatmap analysis of cells isolated from the ischemic areas (Isc) and the SVZ, as well as brain pericytes (PCs) (**P**). Expression levels of Nestin genes were similar between cells from the Isc and those from the SVZ (**P**); however, cells from the SVZ exhibited high expression of neuroepithelial lineage markers such as Pax6 and CD133 (**P**). In contrast, cells from the Isc exhibited high expression of pericytic markers such as NG2, PDGFRβ, CD73, and αSMA, similar to brain PCs (**P**). Results are representative of three replicates (**A**–**O**). Scale bars: 100 µm (**A**,**B**,**D**,**E**) and 50 µm (**G**–**N**). Additional abbreviations: αSMA, alpha smooth muscle actin; DAPI, 4′,6-diamidino-2-phenylindole; GFAP, glial fibrillary acidic protein; GFP, green fluorescent protein; MAG, myelin-associated glycoprotein; MAP2, microtubule-associated protein 2; MBP, myelin basic protein; NF, neurofilament; NG2, neural/glial antigen 2; PAX6, paired box 6; PDGFRβ, platelet derived growth factor receptor-beta; PLP, proteolipid protein; RT-PCR, reverse transcription polymerase chain reaction; SVZ, subventricular zone.

To determine whether GFP^+^ cells developing in the ischemic areas and the SVZ have the traits of NSPCs, we isolated cells from the ischemic areas and the ipsilateral side of SVZ, respectively, and then incubated these cells in adhered cultures to promote the proliferation of NSPCs. Consequently, adhered cells emerged from cell cultures of the ischemic areas (Figure 7A) or the ipsilateral side of the SVZ (Figure 7B). Morphologically, their shapes differed; cells from the ischemic areas had shapes that were more spindle-like compared with the shapes of those from the SVZ (Figure 7A,B).

We then assessed the NSPC activities of the cells. Following incubation using floating cultures (Figure 7C), the formation of neurosphere-like cell clusters was observed from the ischemic areas (Figure 7D) and the ipsilateral side of the SVZ (Figure 7E). According to reverse transcription polymerase chain reaction (RT-PCR), neurosphere-like cell clusters obtained from both areas showed NSPC markers, including *Nestin* and *Sox2* as well as *GFP* (Figure 7F). Following differentiation, the neuronal differentiation potential of GFP^+^ cells was investigated. Immunohistochemistry showed that, although some cells (especially in the areas of spheres) retained GFP expression even after differentiation (Figure 7G,H), differentiated cells from the ischemic areas and the SVZ expressed neuronal markers [Tuj1 (Figure 7G,H) and MAP2 (Figure 7I,J)], an astrocytic marker [GFAP (Figure 7K,L)], and an oligodendrocyte marker [MBP (Figure 7M,N)]. In addition, RT-PCR analysis showed that the differentiated cells obtained from both areas expressed various neural markers including neurons (*Tuj1*, *MAP2*, and *neurofilament*), astrocytes (*GFAP*), and oligodendrocytes (*MBP*, *PLP*, and *MAG*). Notably, strong *GFAP* expression was observed in cells from the SVZ compared with that from cells in the ischemic areas (Figure 7O).

We further compared gene expression patterns of cells from the ischemic areas (Figure 7A) and from the SVZ (Figure 7B) with those from brain pericytes because we had previously shown that cells with NSPC activities originated in part from reactive brain pericytes upon ischemic stimuli [7,10,18]. Heatmapping (Figure 7P) based on microarray analysis showed that, although a similar degree of *Nestin* expression was observed in cells from the ischemic areas and from the SVZ, higher levels of neuroepithelial lineage markers, such as *Pax6* and *Prominin1* (*CD133*), were observed in cells from the SVZ. In addition, signal intensity of pericytic markers, such as *CSPG4* (*NG2*), *PDGFRβ*, *NT5E* (*CD73*), and *ACTA2* (*αSMA*), was higher in cells from the ischemic areas compared with that from cells in the SVZ. Thus, GFP^+^ cells with NSPC activities apparently developed at the ischemic areas and in the SVZ, although these cells likely have diverse origins.

### 2.7. NSPCs at the Site of Ischemic Areas Are Unlikely to Be Derived from NSPCs in the SVZ

**Figure 8:**
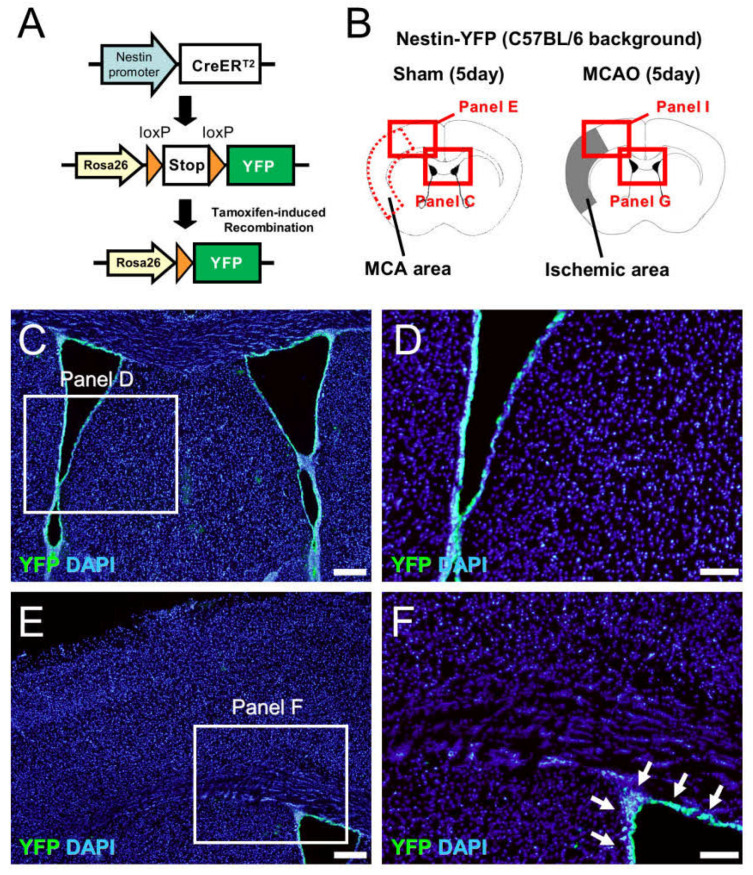

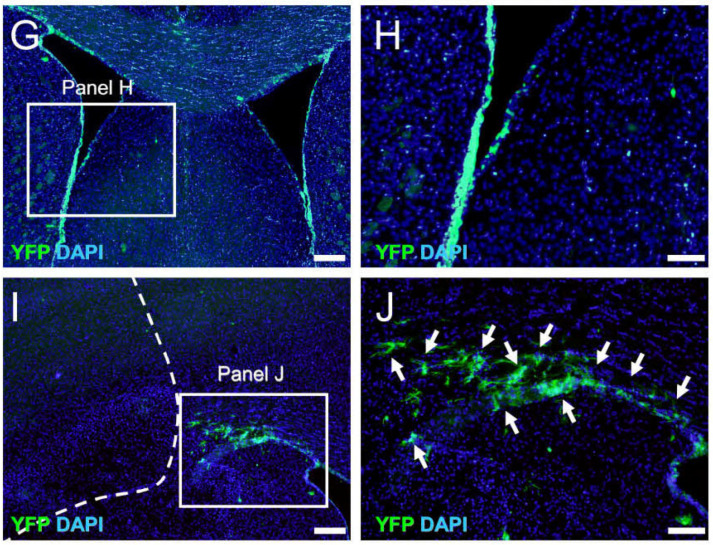
The fate of YFP^+^ NSPCs following MCAO via the Cre-LoxP system (**A**–**J**). Tamoxifen treatment induced recombination in the Rosa26-YFP locus and excision of the stop codon, which resulted in the sustained expression of YFP in cells activated by the Nestin promoter (**A**). Immunohistochemistry for YFP at 5 days after the sham operation (control) (**B**–**F**) and MCAO (**B**,**G**–**J**). YFP^+^ cells were located within the SVZ in sham-operated mice [**C**–**F**, arrows (**F**)]. Although YFP^+^ cells migrated toward the ischemic areas following MCAO, these were not observed within the ischemic areas [**G**–**J**, arrows (**J**)]. [YFP (**C**–**J**: green), DAPI (**C**–**J**: blue)]. Results are representative of three replicates. Scale bars: 200 µm (**C**,**E**,**G**,**I**) and 100 µm (**D**,**F**,**H**,**J**). Abbreviations: NSPC, neural stem/progenitor cell; DAPI, 4′,6-diamidino-2-phenylindole; MCAO, middle cerebral artery occlusion; SVZ, subventricular zone; YFP, yellow fluorescent protein.

Our data indicate that, although GFP^+^ cells isolated from the ischemic areas and the SVZ both have NSPC activities, they also have distinctive traits. Therefore, GFP^+^ cells developing in the ischemic areas are not likely to be derived from the cells in the SVZ. To clarify the relationship between the NSPCs in the ischemic areas and in the SVZ, we performed genetic mapping experiments using the Cre-LoxP system, with which we established a strain of mice expressing yellow fluorescent protein (YFP) under the regulation of the *Nestin* promoter (Figure 8A). On day 5 after the sham operation, YFP was selectively located within the SVZ in sham-operated mice (Figure 8B–F), indicating that these mice are useful for tracing the fate of SVZ-derived NSPCs. On day 5 after ischemic stroke, YFP^+^ cells in the ipsilateral side of the SVZ migrated toward the injured sites following ischemic stroke. However, the migratory capacity of these cells was limited; thus, YFP^+^ cells were not observed within or around the ischemic areas of the cortex (Figure 8B,G–J). Hence, these results strongly suggest that GFP^+^ NSPCs developing at the ischemic areas and in the SVZ have different origins.

## 3. Discussion

This study demonstrated for the first time that the survival rates of Nestin-GFP mice (CB-17 background) are higher than those of Nestin-GFP mice (C57BL/6 background). In addition, infarcted volume was reproducible in Nestin-GFP mice (CB-17 background), which is consistent with the traits of CB-17 wild-type mice [10,11,17].

Traditionally, mice with the C57BL/6 background have been used for studies in many research fields, including in research on the CNS. However, evidence is increasingly showing that this strain of mice has variable ischemic areas following MCAO [17,19,20]. This is due to mice with the C57BL/6 background having many arterial branches around the MCA that cause heterogeneous infarction of various sizes following MCAO. In contrast, mice with the CB-17 background do not show this trait [17]. Therefore, in the present study, we established Nestin-GFP mice (CB-17 background) using backcrossing techniques that have been widely used in various cell biology fields including zoology and botany [21,22,23]. Consistent with the traits of CB-17 wild-type mice [10,11,17], the present results showed that ≥N6 Nestin-GFP mice (CB-17 background) had fewer arterial branches around the MCA compared with Nestin-GFP mice (C57BL/6 background). In addition, following MCAO, Nestin-GFP mice (CB-17 background) showed highly reproducible infarction size, which was limited to the cerebral cortex from the ipsilatereal side of the cortex. In contrast, Nestin-GFP mice (C57BL/6 background) displayed variable sizes of infarcted areas.

Mice with a C57BL/6 background exhibit other critical issues, such as high mortality following MCAO [17]. Consistent with previous findings, the present study found that Nestin-GFP mice (C57BL/6 background) had higher mortality than Nestin-GFP mice (CB-17 background), all of which survived for the 28-day test period after MCAO. Thus, Nestin-GFP mice (CB-17 background) from more than six generations acquired the important traits of CB-17 wild-type mice. Previous studies have shown that C57BL/6 strain mice have a wide range of ischemic areas other than the cerebral cortex, including the striatum [3,17,24,25,26]. The Nestin-GFP mice (C57BL/6 background) in the present study displayed a similar trend for their ischemic areas; therefore, a wide range of brain damage (e.g., ischemia and edema), which frequently occurs in strains with a C57BL/6 background, may be associated with the high mortality observed in these mice.

It is well known that Nestin is expressed in NSPCs in the CNS [27]. Although the precise traits and origin of Nestin^+^ NSPCs in the CNS remain unclear, previous studies using Nestin-GFP mice have shown that Nestin is expressed in various types of stem cell populations including NSPCs in the SVZ and the subgranular zone [28]. During early development, Nestin expression occurs widely across the entire CNS; however, its expression gradually disappears at later stages of development [16]. In adult brains, Nestin is expressed in specific neurogenic zones such as the SVZ and the subgranular zone [15,16,28]. Consistent with these findings, the present study revealed that Nestin was mainly expressed in the SVZ of Nestin-GFP mice under nonischemic conditions.

Following ischemic stroke in the studied mice, Nestin expression was further increased in the ipsilateral side of the SVZ, indicating that ischemia activated endogenous NSPCs in the SVZ. However, although Nestin^+^ NSPCs in the SVZ of Nestin-GFP mice (CB-17 background) migrated toward injured regions after ischemic stroke, they did not likely reach the ischemic areas of the cortex. In contrast, previous studies have shown that NSPCs in the SVZ not only migrate toward the injured regions but also reach the ischemic areas following ischemic stroke [2,3,4]. Notably, however, these studies used mice with a C57BL/6 background [3,4]. Therefore, the ischemic areas are widely distributed outside the cerebral cortex and frequently include the striatum; thus, these areas are closer to the SVZ than to the cerebral cortex. In contrast, in CB-17 mice, the ischemic areas are highly reproducible and are usually restricted within the cerebral cortex at the ipsilatereal side of the cortex [10,11,17]. Therefore, differences in the migratory behavior of SVZ-derived NSPCs following stroke may be related to differences between the ischemic areas of CB-17 strain mice and those of C57BL/6 strain mice.

Using the Cre-LoxP system, we also investigated the fate of Nestin^+^ NSPCs in Nestin-YFP mice. This genetic mapping tool can be used to help understand the fate of Nestin^+^ cells not only in normal early developmental [29,30] and adult brains [31] but also in the injured CNS, including in nonstroke models [29,32]. However, this strain of mice (Nestin-Cre mice) usually has a C57BL/6 background [31,33,34,35]; thus, we could not precisely follow YFP^+^ cells in Nestin-YFP mice following MCAO. Nevertheless, the current study found that Nestin^+^ cells, which were labeled with YFP, were selectively located in the SVZ and did not reach within the ischemic areas, at least until the 5 days following MCAO. These findings indicate that, the during acute period, the Nestin^+^ cells observed within and around the ischemic areas are not derived from the NSPCs in the SVZ, although we cannot completely exclude the possibility that NSPCs in the SVZ reached the ischemic areas at a later time, e.g., during the chronic period. Although the precise origin of SVZ-derived NSPCs remains unclear, previous studies have found that they may be derived from neuroepithelial lineage cells [1]. In support of this hypothesis, microarray analysis in the present study revealed that SVZ-derived NSPCs show high expression of neuroepithelial lineage markers. This analysis also showed that cells with NSPC activities obtained from the ischemic areas expressed more pericytic markers than were expressed by cells from the SVZ. Thus, the current results provide support for those from previous studies, which indicated that the cells are likely derived, in part, from reactive pericytes following ischemic stroke [7,10,18]. Because brain pericytes following ischemia could differentiate into not only neural but also non-neural lineages [12,13,14,18,36,37], GFP^+^ cells from the ischemic areas might have such activities. Nonetheless, the exact traits and origin of Nestin^+^ NSPCs under ischemic conditions should be clarified in further studies.

The *Nestin* gene usually comprises four exons and three introns. The first intron is reportedly related to a non-neural lineage, such as a muscle precursor, whereas the second intron is associated with a neural-specific element [38]. In the current study, we used Nestin-GFP transgenic mice that carried EGFP under the control of the second intron of *Nestin* [15]. We also used Nestin-CreERT2 Line4 mice that modified the second intron of *Nestin* [33] and we produced Nestin-YFP mice via crossing with YFP reporter mice. However, compared with the extent of GFP-expressing Nestin^+^ cells, which were frequently observed in Nestin-GFP mice, YFP-expressing Nestin^+^ cells were observed less frequently within and around the ischemic areas of Nestin-YFP mice. The precise reason for this is unclear; however, it has been reported that, when using the Cre-LoxP system, the expression patterns and localization of Nestin^+^ cells differ depending on the reporter mice [34] and their developmental stage [30]. Thus, YFP may be selectively located at the SVZ rather than at the ischemic areas in the Nestin-YFP mice used in the present study.

## 4. Material and Methods

### 4.1. Animal Studies

All experimental procedures were approved by the Animal Care Committee of the Hyogo College of Medicine (License number: 16-059; 18-061). Adult mice (8–16 weeks-old) were used in all experiments [C57BL/6JJcl mice (Clea Japan Inc., Tokyo, Japan); CB-17/Icr-+/+Jcl mice (Clea Japan Inc.); B6.Cg-Tg(Nes-EGFP)1Yamm mice [15] (RIKEN BioResource Research Center, Ibaraki, Japan); C57BL/6-Tg(Nes-cre/ERT2)4Imayo mice [33] (Nestin-CreERT2 Line4 mice; RIKEN BioResource Research Center); and B6.129-Gt(ROSA)26Sortm1(EYFP)/J mice (YFP reporter mice; Jackson Laboratory, Bar Harbor, ME, USA)]. Nestin-GFP mice provided by the RIKEN BioResource Research Center were produced in relation to the National Bioresource Project of the MEXT/AMED, Japan.

In some experiments, male B6.Cg-Tg(Nes-EGFP)1Yamm mice [Nestin-GFP mice (C57BL/6 background)] were crossed with female CB-17/Icr-+/+Jcl mice (CB-17 wild-type mice). The generated hybrid male mice [CB-17/Icr-+/+Jcl × B6.Cg-Tg(Nes-EGFP)1Yamm] F1 (first filial hybrid) were then continuously backcrossed with female CB-17 wild-type mice. The numbers of backcrossed generations are represented by N [e.g., N2 (second background generation), N3 (third background generation), N4 (fourth background generation), etc.]. Theoretically, hybrid and backcrossed mice should gradually obtain the phenotypes of their counterparts as the number of generations increases [e.g., F1 (50%), N2 (75%), N3 (87.5%), N4 (93.75%), N5 (96.88%), N6 (98.44%), N7 (99.22%), N8 (99.61%), N9 (99.80%), N10 (99.90%), N11 (99.95%), N12 (99.98%), etc.]. Although >N10–N12 is generally recommended for establishing complete congenic mice, >N5 is generally sufficient if early generated hybrids and/or backcrossed mice are used appropriately for further backcrossing [39,40]. To facilitate the backcrossing procedure, among the background hybrid mice for which tail samples were positive for both *Nestin* and *EGFP* genes, we chose mice with skin closer to the white color that is characteristic of CB-17 wild-type mice. During the procedure, the skin of Nestin-GFP mice (C57BL/6 background), which was initially black in color, gradually became gray and then completely white after more than four generations (≥N4). Therefore, we defined mice of more than six generations (≥N6) as Nestin-GFP mice (CB-17 background) and these were used in this study.

Nestin-CreERT2 Line4 mice were crossed with YFP reporter mice and the offspring were termed Nestin promoter-driven YFP-expressing (Nestin-YFP) mice, as previously described [32]. Animals had access to food ad libitum, and efforts were made to minimize the number of animals used and their suffering.

### 4.2. Induction of Ischemic Stroke

Permanent focal cerebral ischemia was produced in mice by ligation and interruption of the distal portion of the left MCA, as previously described [7,8,9,10,11,18,37,41]. In brief, under anesthesia produced by inhalation with isoflurane, mice were placed in a lateral position. Under an operating microscope, an incision was made in the skin and the temporalis muscle was pushed aside to obtain a visual field for craniotomy. A burr hole in the skull was then made using a drill (H021 Minimo; Minitor Co., Ltd., Tokyo, Japan). After opening the dura mater carefully, MCAO was performed using electrocoagulation; this was followed by disconnection of the distal portion of the left MCA. Sham-operated mice underwent the same surgical procedure up until the dura mater was opened.

### 4.3. Tamoxifen Treatment

To activate Cre recombinase, Nestin-YFP mice were treated with tamoxifen (Toronto Research Chemicals Inc., North York, ON, Canada) as previously described [32]. In brief, immediately after recovery following MCAO or the sham operation, tamoxifen (250 mg/kg) was administered using a gastric tube. Nestin^+^ cell fate was then investigated at 5 days poststroke using immunohistochemistry to identify YFP.

### 4.4. Infarct Volume Evaluation

Infarct volume was analyzed after MCAO as previously described [10,11]. Briefly, mice were deeply anesthetized with isoflurane at 1 day (24 h) poststroke. Brains were removed and cut into coronal brain sections (2 mm thickness), after which they were stained with 1% TTC (Sigma-Aldrich, St. Louis, MO, USA) for 20 min at 37 °C in the dark. The sections were then fixed in 4% paraformaldehyde (PFA)/phosphate-buffered saline (pH 7.4). After capturing images of the sections using a microscopic digital camera system (Olympus, Tokyo, Japan), the TTC-unstained region of each slice was calculated using National Institutes of Health Image (Image J) software (Version 1.62) as previously described [9,37,42,43]. The TTC-unstained volume was then measured by multiplying the area by the section thickness. Infarct volume was calculated by summing the values of each slice, as previously described [11].

### 4.5. Preparation of Brain Samples Following Ischemic Stroke

Mice were treated with an intraperitoneal administration (10 mL/kg) of a mixture containing medetomidine (0.3 mg/kg), midazolam (4 mg/kg), and butorphanol (5 mg/kg) [44], and transcardially perfused with 4% PFA as described in earlier studies [7,9,10,11,18,43]. Whole brains were then removed and postfixed with 4% PFA for 24 h. Subsequently, some samples were embedded in paraffin and cut into 8 μm sections. H&E staining was performed against sections obtained from the center of the forebrain. Other fixed brains were cryoprotected in 30% sucrose, frozen at −80 °C, and sliced into 16-μm sections using a cryostat in preparation for immunohistochemistry.

### 4.6. Immunohistochemistry

Brain sections (16-μm thickness) obtained from Nestin-GFP mice were subjected to immunohistochemistry using primary antibodies against GFP [1:1000, rabbit, Abcam (ab6556), Cambridge, UK; 1:2000, chicken, Abcam (ab13970)], GFAP (1:500, mouse, Millipore, Billerica, MA, USA), NG2 (1:500, rabbit, Millipore), and PDGFRβ (1:500, goat, R&D systems, Minneapolis, MN, USA). To detect YFP^+^ cells in the brain sections of Nestin-YFP mice, an anti-GFP antibody [1:1000, rabbit, Abcam (ab6556)] that recognizes YFP was used. Bound primary antibodies were visualized using Alexa Fluor 488—or 555-conjugated secondary antibodies (1:500, Molecular Probes, Eugene, OR, USA). Nuclei were counterstained with 4′,6-diamidino-2-phenylindole (DAPI; 1:500, Kirkegaard & Perry Laboratories, Inc., Gaithersburg, MD, USA). Images were captured using a confocal laser microscope (LSM780; Carl Zeiss AG, Oberkochen, Germany) and a microscope (Olympus, Tokyo, Japan).

### 4.7. Cell Cultures

To investigate whether GFP^+^ cells developing in the ischemic areas have NSPC activities, tissues were carefully removed from the ischemic areas at 3 days poststroke. The removed tissues were then mechanically dissociated by passaging through 18-, 23-, and 27-gage needles, and the resulting single-cell suspension was incubated using adhered cultures in Dulbecco’s modified Eagle medium/F12 (Thermo Fisher Scientific, Rochester, NY, USA) containing 20 ng/mL of basic fibroblast growth factor (bFGF; PeproTech, Rocky Hill, NJ, USA), 20 ng/mL of epidermal growth factor (PeproTech), 1% N_2_ supplement (Invitrogen, Carlsbad, CA, USA), and 2% fetal bovine serum (FBS) to promote the proliferation of NSPCs, as previously described [10,11,18,32]. When adhered cells reached confluence, they were detached with Accutase. Subsequently, they were subjected to floating cultures in medium that accelerated the formation of neurosphere-like cell clusters. On day 14 after incubation, neurosphere-like cell clusters were collected, subjected to RT-PCR, and differentiated on poly-L-lysine-coated glass coverslips for 10 days in neurobasal medium (Invitrogen) containing bFGF, B-27 supplement (Invitrogen), and 2% FBS, as previously described [11,32]. The differentiated cell clusters were also subjected to RT-PCR as well as immunohistochemistry using antibodies against GFP [1:1000, rabbit, Abcam (ab6556); 1:2000, chicken, Abcam (ab13970)], Tuj1 (1:1000, mouse, Stemcell Technologies, Vancouver, BC, Canada), MAP-2 (1:1000, rabbit, Millipore), GFAP (1:1000, rabbit, Abcam), and MBP (1:100, mouse, R&D systems). Bound primary antibodies were visualized using Alexa Fluor 488—or 555-conjugated secondary antibodies (1:500; Molecular Probes).

### 4.8. Reverse Transcription Polymerase Chain Reaction

Total RNA was extracted using an RNeasy Micro Kit (Qiagen, Hilden, Germany) as described in earlier work [11,12,13,18,32]. Briefly, total RNA was extracted from cell clusters or differentiated cell clusters obtained from the ischemic areas and the SVZ. Subsequently, cDNA was amplified according to the manufacturer’s protocol. The primer sequences used in this study are listed in Table 1.

### 4.9. Microarray Analysis

Total RNA was isolated from the adhered cells obtained from the ischemic areas and the SVZ using an RNeasy Micro Kit (Qiagen). In addition, total RNA was extracted from commercially available mouse brain pericytes (#M1200, Scien Cell Research Laboratories) that were maintained in medium according to the manufacturer’s protocol. Samples were then subjected to microarray analysis as previously described [13,14,36]. Briefly, microarray experiments consisting of RNA quality assessment, labeling, hybridization, scanning, and data analysis were conducted via a contract service (Takara Bio Inc., Shiga, Japan).

### 4.10. Statistical Analysis

Data are presented as means ± standard deviations. Statistical comparisons among multiple groups were determined using one-way ANOVA followed by Bonferroni post-hoc tests. Where indicated, comparisons were performed using Student’s *t*-test. The survival rate was analyzed using a logrank test. *p* values of <0.05 were considered statistically significant.

## 5. Conclusions

In conclusion, we found that Nestin-GFP mice (C57BL/6 background) could acquire the phenotypes of Nestin-GFP mice (CB-17 background) via a process of backcrossing with CB-17 wild-type mice. We also found that, compared with Nestin-GFP mice (C57BL/6 background), Nestin-GFP mice (CB-17 background) exhibited reproducible ischemic areas and higher survival rates following MCAO. Therefore, Nestin-GFP mice (CB-17 background) could be used to investigate the precise roles of NSPCs and the NSPC-related mechanism of neurogenesis over long periods after ischemic stroke.

## Figures and Tables

**Table 1:** List and sequences of mouse primers used for RT-PCR analysis.

Primers	Sequence (5′ → 3′) (F: Forward; R: Reverse)	Size
β-actin	F:GCTCGTCGTCGACAAGGGCTC; R:CAAACATGATCTGGGTCATCTTCTC	353 bp
GFAP	F:TCGGCCAGTTACCAGGAGG; R:ATGGTGATGCGGTTTTCTTCG	176 bp
GFP	F:ATCATGGCCGACAAGCAGAAGAAC; R:GTACAGCTCGTCCATGCCGAGAGT	258 bp
MAG	F:CAAGTCCCGCACACAAGTG; R:AGCAGGGTACAGTTTCGTAGG	87 bp
MAP2	F:CTCATTCGCTGAGCCTTTAGAC; R:ACTGGAGGCAACTTTTCTCCT	159 bp
MBP	F:TCACAGCGATCCAAGTACCTG; R:CCCCTGTCACCGCTAAAGAA	125 bp
nestin	F:CGCTGGAACAGAGATTGGAAG; R:CATCTTGAGGTGTGCCAGTT	158 bp
NF	F:CCGTACTTTTCGACCTCCTACA; R:CTTGTGTGCGGATAGACTTGAG	247 bp
PLP	F:TGAGCGCAACGGTAACAGG; R:GGGAGAACACCATACATTCTGG	295 bp
Sox2	F:TTGGGAGGGGTGCAAAAAGA; R:CCTGCGAAGCGCCTAACGTA	312 bp
Tuj1	F:TGAGGCCTCCTCTCACAAGT; R:GGCCTGAATAGGTGTCCAAA	105 bp

## Data Availability

The data supporting the findings of this study are available within the article.

## Supplementary Materials

The following are available online at https://www.mdpi.com/article/10.3390/ijms222312997/s1.
