# Supplementary material for: Establishment of a Reproducible Ischemic Stroke Model in Nestin-GFP Mice with High Survival Rates
Source: Int J Mol Sci. 2021 Nov 30;22(23):12997. doi: 10.3390/ijms222312997 (PMC8657611; doi:10.3390/ijms222312997)
Supplement: Supplementary file 1 [file ijms-22-12997-s001.zip › ijms-1445019-Supplementary.pdf]

Supplementary Figure Legend

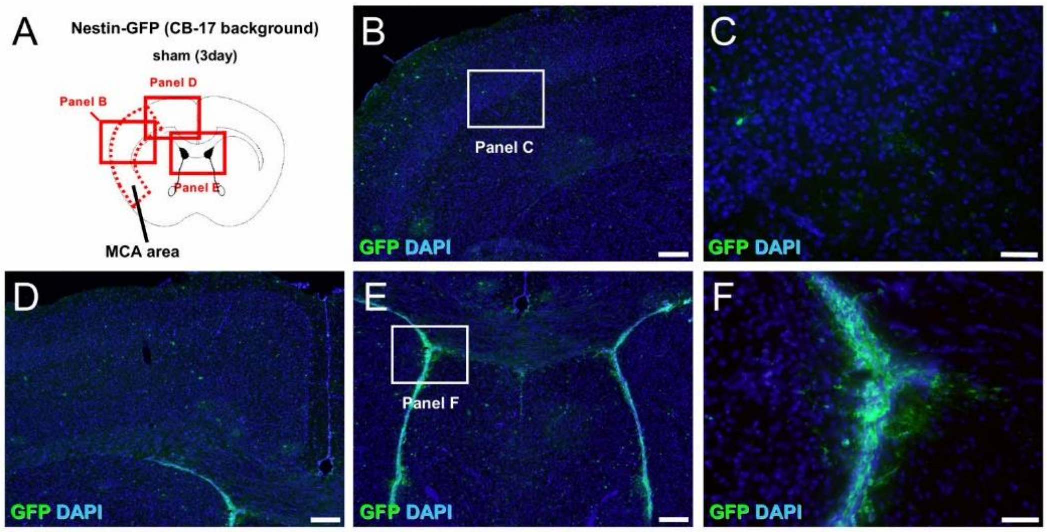

Supplementary Figure S1: Immunohistochemistry for GFP at 3 days after sham-operation (**A–F**). GFP was rarely observed within and around the MCA areas of cortex (**B-D**), whereas GFP was strongly observed in the SVZ (**E, F**). [GFP (**B-F**: green), DAPI (**B-F**: blue)]. Results represent three replicates. Scale bars: 200 µm (**B, D, E**) and 50 µm (**C, F**). Abbreviations: DAPI, 4',6-diamidino-2-phenylindole; GFP, green fluorescent protein; MCA, middle cerebral artery; SVZ, subventricular zone.

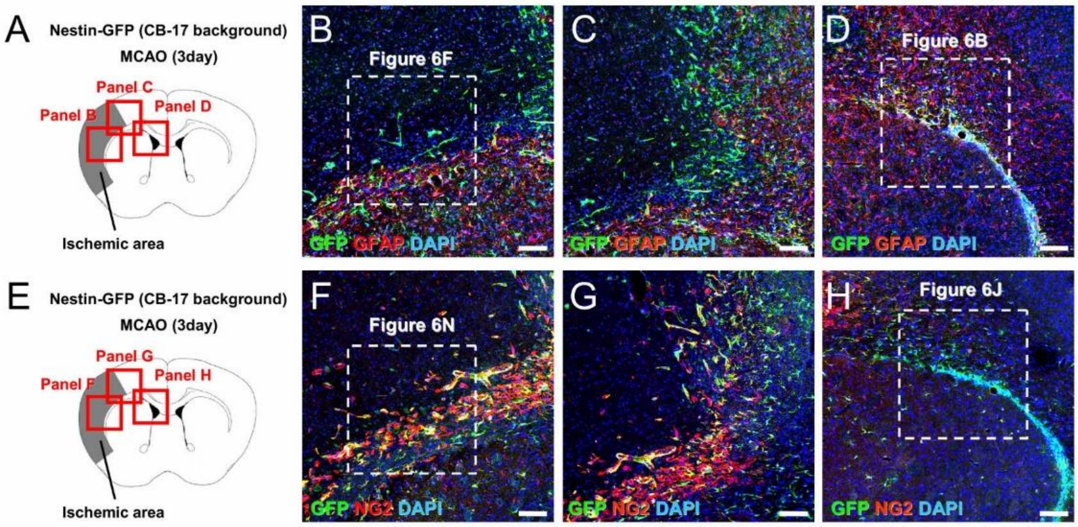

Supplementary Figure S2: Double immunohistochemistry for GFP/GFAP (**A–D**) and GFP/NG2 (**E–H**) at the ischemic sites (**B, C, F, G**) and the SVZ (**D, H**) at 3 days after MCAO [GFP (**B–D, F–H**: green), GFAP (**B–D**: red), NG2 (**F–H**: red), and DAPI (**B–D, F–H**: blue)]. Results represent three replicates. Scale bars: 100 µm (**B, C, D, F, G, and H**). Abbreviations: DAPI, 4',6-diamidino-2-phenylindole; GFAP, glial fibrillary acidic protein; GFP, green fluorescent protein; MCAO, middle cerebral artery occlusion; NG2, neural/glial antigen 2; SVZ, subventricular zone.
